# Essential competencies of nurses for climate change response in Saudi Arabia: A rapid literature review

**DOI:** 10.1111/jan.16372

**Published:** 2024-08-06

**Authors:** Zakaria A. Mani, Katarzyna Naylor, Krzysztof Goniewicz

**Affiliations:** ^1^ Nursing College Jazan University Jazan Saudi Arabia; ^2^ Faculty of Medicine, Nursing and Health Science Monash University Melbourne Victoria Australia; ^3^ Department of Emergency Medicine Medical University of Lublin Lublin Poland; ^4^ Department of Security Polish Air Force University Deblin Poland

**Keywords:** climate change, cultural considerations, disaster preparedness, nurses, Saudi Arabia, vulnerable populations

## Abstract

**Aim:**

Amidst the mounting challenges posed by climate change, the healthcare sector emerges as a vital frontliner, with nurses standing as its linchpins. This review delves into the pivotal role of nurses in combatting the health consequences of climatic alterations, particularly within the nuanced environment of Saudi Arabia.

**Design:**

A rapid literature review.

**Method:**

Drawing from a rigorous analysis of 53 studies, our exploration revolves around the preparedness strategies formulated in response to Saudi Arabia's changing climate. The variables analysed included study design, sample size, focus area, geographical coverage and key findings related to nurse competencies. Data were collected using a structured data extraction form and analysed using thematic content analysis. Employing content analysis, we discerned essential domains: from grasping the health impacts of climate change to customizing care for the most susceptible populations and championing advocacy initiatives.

**Findings:**

Salient findings highlight nurses' profound understanding of both direct and secondary health implications of climate shifts. Additionally, the results emphasize the tailored interventions needed for vulnerable groups, capacity building and disaster readiness. Crucially, our findings spotlight the significance of weaving cultural, ethical and regional threads into nursing strategies. By painting a comprehensive picture, we showcase the delicate balance of environmental evolution, healthcare dynamics and the unique socio‐cultural tapestry of Saudi Arabia.

**Conclusion:**

The results of our analysis revealed key competencies required for nurses, including the ability to address immediate health impacts, provide tailored care for vulnerable populations and engage in advocacy and policy formulation. In summation, nurses' multifaceted roles—from immediate medical care to research, advocacy and strategizing—underscore their invaluable contribution to confronting the health adversities sparked by climate change. Our review accentuates the essential contributions of nurses in tackling climate‐related health hurdles and calls for more nuanced research, policy adjustments and proactive measures attuned to Saudi Arabia's distinct backdrop.

## 1 INTRODUCTION

Climate change, once considered an abstract concept or a distant future concern, has swiftly emerged as one of the most significant challenges of the 21st century. This global crisis impacts various aspects of human life, from economies to health, affecting every corner of the world, including the Middle East and countries like Saudi Arabia (Almulhim & Cobbinah, 2023).

Nursing, inherently a profession rooted in care, empathy and adaptability, finds itself at the intersection of healthcare and climate science. With an escalating frequency of climate‐induced health emergencies, the profession demands nurses who are not just clinically competent but also environmentally conscious (Richards et al., 2023). In essence, nursing in the age of climate change necessitates a paradigm shift—a move from traditional care models to more dynamic, responsive and holistic care paradigms that factor in environmental realities (Nicholas et al., 2020).

Nurses, being at the forefront of healthcare, play a pivotal role in responding to the health challenges posed by climate change. Their competencies and skills are crucial in ensuring effective care delivery in an environment that's continually being reshaped by climatic shifts (Goniewicz, Khorram‐Manesh, Włoszczak‐Szubzda, et al., 2023). However, with this ever‐evolving scenario, there's a palpable gap in our understanding of the core competencies required by nurses, especially in specific contexts like Saudi Arabia, to respond adeptly to these changes.

The diverse and intricate landscape of Saudi Arabia, spanning deserts, coasts and urban conglomerates, brings forth unique challenges. Climatic extremes, be it scorching heatwaves or sudden flash floods, have direct and indirect repercussions on community health. These range from heat‐related illnesses and waterborne diseases to more indirect consequences like displaced populations seeking healthcare (Mani & Goniewicz, 2023). Furthermore, there is a significant inequality in the impact of climate change among different populations, with vulnerable groups such as the socio‐economically disadvantaged, disabled individuals and remote communities facing heightened risks (Mahmoud et al., 2023).

Efforts have been made globally and regionally to address these issues, including integrating climate‐related health challenges into healthcare curricula and training. However, Saudi Arabia still has ground to cover in tailoring these solutions to its unique socio‐cultural and climatic context (Almutairi et al., 2019). This lack of tailored research and solutions specific to the Saudi context underscores the urgency of our investigation.

Our research aims to bridge this gap by identifying the essential healthcare nurse competencies required for an effective climate change response in Saudi Arabia. By systematically exploring these competencies and proposing specific training and educational interventions, we offer a novel framework that addresses both global best practices and regional specificities.

Thus, the question arises: What are the essential healthcare nurse competencies required for an effective climate change response in Saudi Arabia? And how can these competencies be instilled, honed and applied in the Saudi healthcare context? Answering these questions is not merely an academic exercise but a critical step towards future‐proofing the Saudi healthcare system and ensuring that its nurses are equipped to protect and promote health in the face of climate‐related challenges.

A holistic understanding of this topic extends beyond the confines of clinical practice. It dives into socio‐cultural dynamics, healthcare policies, infrastructure readiness and even geopolitical considerations. Saudi Arabia, as a key player in the global energy market and a nation undergoing significant socio‐economic reforms, has a unique position in the global climate narrative (Dargin, 2021). Its strategies, policies and on‐ground actions in response to climate change have broader implications for global health and climate diplomacy (Dargin, 2021; Tagliapietra, 2019).

The global readership must recognize the significance of this research. While centred on Saudi Arabia, the findings and discussions can provide a blueprint for other countries, especially those with similar socio‐cultural and climatic profiles. The insights can guide global health policy decisions, curricula development and capacity‐building initiatives.

While the focus of this research is predominantly on Saudi Arabia, its implications resonate on a much broader scale. Climate change knows no boundaries, and its effects are felt universally, albeit with regional variations (Lyon et al., 2022; Nwankwo et al., 2020; Parmesan et al., 2022). As such, understanding the healthcare response to these challenges in one region can offer invaluable insights for others. The lessons derived from Saudi Arabia's unique geographical, socio‐cultural and healthcare contexts can serve as exemplars or cautionary tales for other nations in their journey to integrate climate science into healthcare delivery. Moreover, the collaborative spirit of the global health community necessitates the sharing of knowledge, insights and best practices (Cajete, 2020; Marselle et al., 2019). Through this research, we hope to contribute to a shared global repository of knowledge, ensuring that strategies employed in one region can be adapted, adopted and refined in another, always with an eye on regional specificity and cultural appropriateness.

Furthermore, our journey through this research landscape highlights the intricate balance between global commonalities and regional specificities. While the overarching themes of climate change and health impacts might echo global patterns, the nuances of nurse competencies required in the Saudi context shed light on the importance of culturally and regionally aligned healthcare strategies.

It is also imperative to underscore that the competencies discussed herein are not static. As the ramifications of climate change evolve and as our understanding deepens, so must the skillsets of healthcare professionals (Porter et al., 2020). Continuous learning, adaptability and a commitment to interdisciplinary collaboration become vital pillars for modern nursing (Shrivastava et al., 2020). The dynamic nature of climate change demands a healthcare workforce that is not only responsive but also proactive, anticipating challenges and preparing in advance to address them (Eilam, 2022).

The role of educational institutions, nursing associations and policymakers cannot be understated in this transformative journey. By integrating climate literacy and environment‐centric modules into nursing curricula, we can foster a new generation of nurses who view health through the lens of ecology, understanding the inextricable link between human well‐being and environmental stability (Rehman et al., 2021).

Additionally, the evolving digital landscape offers a plethora of opportunities. Digital health tools, telemedicine and AI‐driven predictive analytics can play a pivotal role in enhancing the preparedness and response of nurses to climate‐related health challenges. Embracing technology, while staying rooted in the foundational ethos of nursing, can pave the way for a more resilient healthcare system (Rosa et al., 2019).

Saudi Arabia, with its Vision 2030 and other transformative initiatives, is at a critical juncture (Chowdhury et al., 2021). The nation's commitment to diversifying its economy and enhancing its socio‐cultural fabric aligns seamlessly with the need to strengthen its healthcare sector's response to climate change. Nursing, as a profession, stands to play a monumental role in this transformation.

The essence of this research is not just to identify the gaps but to pave pathways for bridging them. The aim of this study is to explore, elucidate and enumerate the essential healthcare nurse competencies for an effective response to climate change in Saudi Arabia. By comprehensively understanding the competencies required by nurses in the context of climate change, especially in Saudi Arabia, we aim to contribute to the larger global discourse on health, environment and sustainable development (Bajow et al., 2022). Through a thorough review of the literature, we endeavour to provide a comprehensive framework that can serve as a foundation for future research, policy formulation and curricular enhancements in the realm of nursing and climate change, both within Saudi Arabia and globally.

## 2 MATERIALS AND METHODS

In this rapid review, we aimed to efficiently synthesize literature highlighting the essential competencies of nurses in addressing climate change challenges, specifically in the Saudi Arabian context. We adopted a streamlined approach, informed by a precise protocol, to ensure swift yet robust data gathering. The decision to conduct a rapid review was driven by the urgent need to understand the essential competencies of nurses in addressing climate change challenges in Saudi Arabia. Given the escalating impacts of climate change on health and the immediate need for evidence‐based interventions, a rapid review allows for a timely synthesis of the available literature. This approach balances comprehensiveness and expediency, enabling us to provide relevant insights within a shorter timeframe compared to a full systematic review. The constraints of time and resources in addressing an emerging and pressing issue such as climate change necessitated this approach, ensuring that healthcare professionals can access and implement the findings without undue delay.

### 2.1 Type of research and design

This study is a rapid review, a type of research that involves a streamlined approach to synthesizing evidence in a timely manner. The design of the study is a systematic review, which involves systematically searching, selecting and analysing relevant literature to answer specific research questions. The variables collected include study design, sample size, focus area, geographical coverage and key findings related to nurse competencies in responding to climate change.

### 2.2 Search strategy

Our comprehensive search strategy involved a systematic review of the literature. We searched our comprehensive search strategy involved a systematic review of the literature. We searched three primary electronic databases: PubMed, Scopus and Web of Science. The search was limited to studies published between 2010 and 2023. The keywords used included ‘nurse competencies’, ‘climate change response’ and ‘Saudi Arabia’. Each database was searched with a specific algorithm detailed in our protocol.

### 2.3 Criteria for literary inclusion

We sought English‐language articles that had undergone peer review and discussed the intersection of nurse competencies and climate change within Saudi Arabia. Studies that were editorials, standalone abstracts or unrelated to the topic were excluded.

### 2.4 Methodology for selecting studies

After removing duplicates, a single experienced reviewer screened titles and abstracts. To address potential biases and oversight, a second reviewer was consulted in instances of uncertainty. Selected articles underwent a full‐text review, emphasizing the unique Saudi context.

### 2.5 Nature and scope of chosen studies

We identified and reviewed a total of 53 studies that met our inclusion criteria. These studies utilized diverse methodologies, including qualitative insights, quantitative analyses and literature reviews. Key characteristics of the included studies are summarized as follows: the study designs ranged from cross‐sectional, longitudinal and case studies; sample sizes varied from small qualitative samples to large‐scale surveys; focus areas included health impacts of climate change, nurse preparedness, vulnerability assessments and intervention strategies; and the regions covered various areas within Saudi Arabia, highlighting the geographic and climatic diversity of the country.

### 2.6 Instruments used for data collection

To extract data, we used a data extraction form specifically designed for this review. The form included fields for study design, sample size, focus area, key findings and geographical coverage. Additionally, we employed a thematic analysis approach to identify recurring patterns and narratives in the data.

### 2.7 Efficient data extraction and theme identification

Using our protocol, we extracted essential data from each article, focusing on study objectives, methodologies, key findings and implications. A thematic analysis was performed to pinpoint recurring patterns and narratives.

### 2.8 Streamlined research integrity: Quality evaluation

In the interest of time and the rapid nature of this review, we utilized an informal yet structured approach to assess the quality of the selected articles. While we initially intended to employ the Critical Appraisal Skills Programme (CASP) tool, the time constraints necessitated a more streamlined assessment process. We focused on key aspects of quality, such as study design, sample size and relevance to the research questions, to quickly identify the strengths and limitations of each article. This approach allowed us to efficiently guide our selection process while ensuring that the core quality indicators were still considered.

### 2.9 Rapid data synthesis

Extracted insights were synthesized using a narrative approach. We organized data into thematic groups, facilitating deeper understanding and interpretation.

### 2.10 Resolving disagreements

Given the condensed nature of our review, disagreements among team members were resolved through open dialogue. On rare occasions, an external expert was consulted to provide clarity.

## 3 RESULTS

**FIGURE 1:**
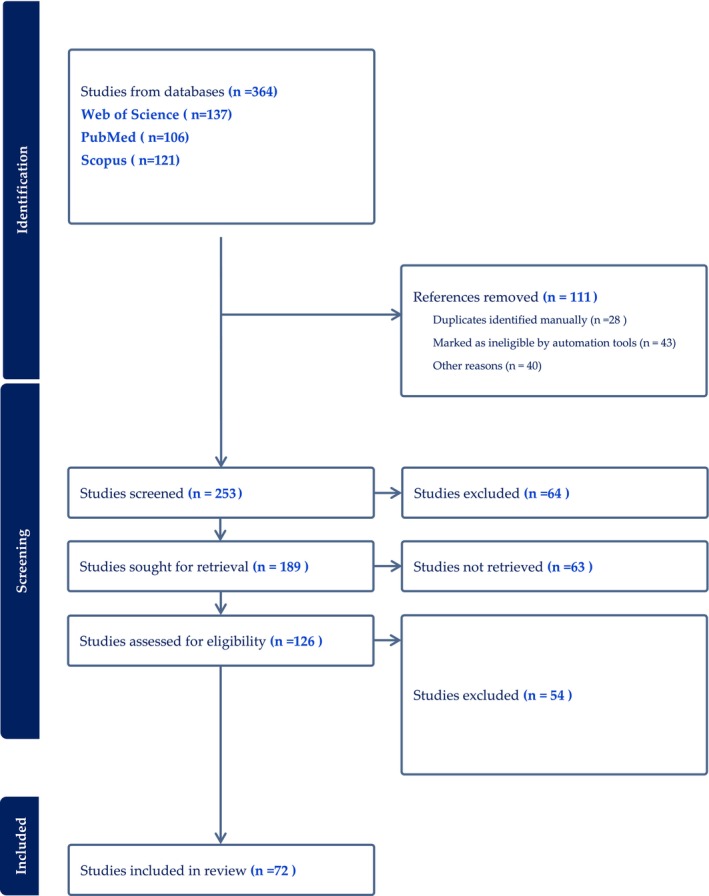
Flow Diagram of Literature Selection Process.

From our meticulous search, we assessed a total of 67 studies (Figure 1) (Moher et al., 2009), offering invaluable insights into the critical competencies nurses must foster in light of the impending and ongoing climate shifts, particularly in the Saudi Arabian milieu.

To provide a clearer overview of our findings, we have summarized the key competencies identified in these studies in Table 1. This table highlights the core domains, specific competencies and key points that emerged from our review, setting the stage for a more detailed discussion in the subsequent sections.

**TABLE 1:** Summary of core competencies and key findings.

Core domain	Specific competencies	Key points
Understanding of climate change and its health impacts	Foundational knowledge on direct and indirect health impacts	Importance of recognizing immediate and long‐term health consequences; unique climatic challenges in Saudi Arabia
Direct and indirect health impacts with nursing interventions	Acute care and community education	Immediate response to heatwaves and storms; public health campaigns for respiratory and waterborne diseases
Vulnerable population care	Tailored interventions for socio‐economically disadvantaged, disabled, pregnant women, remote communities, children and older adults	Focus on building community resilience, inclusive disaster preparedness and age‐specific care
Capacity building, education and advocacy	Training healthcare workers, community outreach, policy advocacy	Empowerment through continuous education; integrating technology for training and advocacy
Disaster preparedness and emergency response	Anticipation, preparation and response to climate‐induced emergencies	Skills in triage, mass casualty management, use of technology like GIS and mobile health apps for disaster response
Research, adaptation and evidence‐based practice	Conducting and integrating research into practice	Leveraging digital health tools, interdisciplinary collaborations, and adapting global practices to local contexts
Cultural, ethical and regional csonsiderations	Providing culturally competent and ethically sound care	Understanding local traditions and beliefs; addressing health disparities and ethical dilemmas related to resource allocation

The subsequent sections provide a comprehensive analysis of these core domains and competencies. We delve into the intricate details, exploring the progress made to date and highlighting the imminent challenges and opportunities for further development. By examining these aspects, we present a thorough understanding of the essential competencies required for nurses to effectively address climate change impacts, tailored to the unique socio‐cultural and environmental context of Saudi Arabia.

### 3.1 Understanding of climate change and its health impacts

The recognition of climate change's profound effects on global health has underlined the urgency to equip healthcare professionals, especially nurses, with foundational knowledge on the subject. Within this framework, it's paramount for nurses to possess a comprehensive understanding of both the direct and indirect impacts of climate change, particularly in regions like Saudi Arabia, which faces unique climatic challenges.

Direct impacts, such as heatwaves and storms, can have immediate health consequences. Heatwaves, for instance, can lead to heat‐related illnesses, exacerbate pre‐existing health conditions and even result in fatalities (Vanos et al., 2020). Storms, on the other hand, not only pose immediate threats from physical injuries but also engender long‐term health problems due to the disruption of healthcare services, contamination of water sources and spread of waterborne diseases (McFarland, 2019).

The geographical and climatic idiosyncrasies of Saudi Arabia further intensify these challenges. The nation's arid environment, coupled with its rapidly growing urban areas, creates microclimates that can amplify the severity of heatwaves. This not only increases the physical strain on individuals but can strain the existing healthcare infrastructure, necessitating improved preparedness and response mechanisms (Farahat, 2016).

Equally concerning are the indirect health implications. Alterations in air and water quality can catalyse a plethora of respiratory and waterborne illnesses. A decline in air quality, influenced by increasing temperatures and altered precipitation patterns, can exacerbate respiratory conditions such as asthma and increase susceptibility to respiratory infections (Farahat, 2016). Similarly, deteriorating water quality can increase the spread of diseases like cholera and dysentery, especially in areas with limited access to clean water (Alshareef, n.d.).

Beyond the immediate health implications, there are mental health ramifications of climate change, which often remain underexplored. The stress and anxiety stemming from recurring extreme weather events, coupled with the socio‐economic challenges they engender, can result in psychological distress, depression and other mental health disorders (Alshammari et al., 2022). Nurses, given their frontline role in healthcare, need to be equipped with the skills to recognize and address these psychological manifestations in their patients (Mani & Ibrahim, 2017).

Moreover, ecosystem changes, another indirect effect of climate change, have broad health implications. These transformations can promote the spread of vector‐borne diseases, as changing climates can expand the habitats of vectors like mosquitoes, thus increasing the risk of diseases such as malaria and dengue (Mani & Goniewicz, 2023; Samy et al., 2016).

Saudi Arabia's diverse ecosystem, from its coastal regions to its desert interiors, means that the range of potential vector‐borne diseases varies widely across the nation. Understanding these regional disparities in disease prevalence and transmission is pivotal for nurses, enabling them to provide targeted care and preventive guidance to the communities they serve (Bellizzi et al., 2020).

It is essential for nurses in Saudi Arabia to holistically understand the multifaceted health challenges posed by climate change. Such knowledge is a precursor to effective interventions, patient education and the development of resilience strategies, ensuring that healthcare remains adaptive and responsive in an era of unprecedented environmental change (Mani, 2016; Mani et al., 2023).

### 3.2 Direct and indirect health impacts with nursing interventions

The nexus of climate change and health creates a tapestry of both direct and indirect health challenges. However, with these challenges come opportunities for nurses to spearhead interventions that mitigate the adverse impacts and promote community resilience.

Direct health impacts, such as those from heatwaves, require immediate nursing responses. The acute manifestations of heat‐related illnesses—dehydration, heatstroke and heat exhaustion—demand prompt recognition and action. Nurses play an instrumental role here, not only in administering timely care but also in educating communities about preventative measures such as hydration, appropriate clothing and recognizing early symptoms (Smith et al., 2021).

Storms and associated injuries are another direct outcome of changing climatic patterns. In the aftermath of severe storms, nurses often find themselves at the forefront of emergency response, attending to injuries, administering first aid and coordinating care with other healthcare professionals (Black, 2024). Their role extends beyond the immediate aftermath, as they ensure continuity of care for injured patients, facilitate vaccinations to prevent disease outbreaks and liaise with mental health professionals to address trauma and related psychological manifestations (Zhang et al., 2020).

The indirect health impacts of climate change present a more complex interplay. Deteriorating air quality, as influenced by rising temperatures and altered weather patterns, can exacerbate respiratory conditions. Here, nurses are pivotal in monitoring vulnerable populations, such as asthmatics or the elderly, ensuring they adhere to medication regimens and advising on minimizing exposure during peak pollution periods (Doenges et al., 2022).

Waterborne diseases, another indirect health consequence, demand community‐level interventions. Nurses, given their trusted status in communities, can lead public health campaigns emphasizing the importance of clean water, sanitation and personal hygiene (World Health Organization, 2018). By organizing workshops and information sessions, they can empower communities with knowledge about the prevention and early recognition of diseases like cholera and dysentery (World Health Organization, 2018).

Ecosystem‐driven changes, which can foster the proliferation of vector‐borne diseases, necessitate nursing interventions at both the micro and macro levels (Benis et al., 2021). At the micro level, nurses can aid in early detection and management of diseases like malaria or dengue, ensuring patients receive timely treatment. Simultaneously, at the macro level, they can collaborate with public health departments in vector control programs, community education and the rollout of preventive measures like bed nets or repellents (Benis et al., 2021).

A critical component of nursing interventions lies in capacity building and continuous education. By staying abreast of the latest research and findings related to climate change and health, nurses can ensure that their interventions are evidence‐based and contemporary. Moreover, their active engagement in multidisciplinary teams can promote a holistic and integrated approach to healthcare, ensuring that both the direct and indirect health impacts of climate change are addressed comprehensively (Fukada, 2018).

The intertwined challenges of climate change and health, while daunting, present an opportunity for nurses to reaffirm their role as healthcare leaders (Harrington, 2021). By aligning their interventions with the evolving health landscape, nurses can ensure that communities remain resilient, informed and equipped to face the health challenges of the future.

### 3.3 Vulnerable population care

In the intricate weave of climate change and its repercussions, certain population groups are distinctly more susceptible to adverse health effects. These vulnerabilities stem from a range of factors—socio‐economic, physiological, geographic or a combination thereof. Addressing the unique needs of these groups demands a specialized set of nursing skills, rooted in both empathy and evidence‐based care.

Socio‐economically disadvantaged individuals often bear a disproportionate brunt of the health challenges posed by climate change. Their limited access to resources and healthcare facilities exacerbates health risks, be it from heatwaves or deteriorating air quality (Smith & Sinkford, 2022). Nurses working with these populations focus not just on direct care but also on building community resilience. By fostering local health networks, initiating community‐based surveillance systems and tailoring health education to be culturally and contextually relevant, nurses can make substantial strides in bridging health disparities (Pounder, 2022).

Individuals with disabilities, another vulnerable cohort, require nuanced care. For instance, mobility challenges might hinder their evacuation during severe weather events, or sensory disabilities might limit their ability to access crucial health information. Nurses play a pivotal role in creating inclusive disaster preparedness plans, ensuring that facilities are accessible and that health communication caters to all, regardless of their disabilities (Goniewicz et al., 2021).

Pregnant women, bearing the dual responsibility of their health and that of their unborn child, stand at a heightened risk during climate‐induced events. Nutritional deficiencies, exposure to pollutants or stress from extreme weather can adversely affect maternal and foetal health. Nurses, with their specialized obstetric skills, are often the primary caregivers ensuring that pregnant women receive tailored advice, necessary supplements and psychological support during these challenging times (Wilson et al., 2021).

Remote communities, by virtue of their geographic isolation, face amplified health challenges. Limited infrastructure and healthcare access combined with unique local climate effects require nurses to be adept in both primary healthcare and emergency medicine. Telemedicine, community health worker training and mobile clinics are among the innovative solutions spearheaded by nurses to ensure that remote populations aren't left behind (Al‐Wathinani, Alhallaf, et al., 2023).

Children and older adults, representing both ends of the age spectrum, possess physiological vulnerabilities to climate change. While children's developing bodies and immune systems might make them susceptible to infectious diseases or respiratory issues, older adults could suffer from exacerbations of chronic conditions due to climate stressors. Nurses, specializing in paediatric and geriatric care, respectively, ensure that these age‐specific needs are addressed. From age‐appropriate health education to medication management, their interventions are tailored to protect these vulnerable age groups (Al‐Wathinani, Barten, et al., 2023).

Empowerment is a recurring theme in vulnerable population care. By equipping these populations with the knowledge, tools and resources they need, nurses not only address immediate health challenges but also pave the way for long‐term resilience. Collaborative efforts, involving local communities, policymakers and multidisciplinary health teams, amplify the impact of these interventions, ensuring that the most vulnerable aren't just cared for but are also empowered to care for themselves (Alburaidi et al., 2023).

### 3.4 Capacity building, education and advocacy

In the overarching narrative of climate change and its health implications, the role of capacity building, education and advocacy emerges as pivotal. Nurses, standing at the crossroads of healthcare delivery and community interaction, are uniquely positioned to lead efforts in these domains, shaping both immediate responses and long‐term strategies.

Capacity building entails the empowerment of healthcare workers, fortifying them with the knowledge, tools and resources required to combat climate change‐induced health challenges. This goes beyond mere dissemination of information; it involves hands‐on training, simulations and real‐time feedback. Nurses often spearhead such initiatives, designing and implementing training modules tailored to the local health landscape. They collaborate with academic institutions, ensuring that curricula remain updated with the latest research and best practices in climate health. Furthermore, nurse‐led workshops and training sessions play a pivotal role in ensuring that frontline healthcare workers, especially those in remote or resource‐limited settings, are adequately equipped to tackle emerging health threats (Al‐Hanawi et al., 2019).

Importantly, technology has emerged as an ally in these capacity‐building endeavours. Through the integration of digital platforms, nurses have facilitated remote training sessions, bridging the geographical divide and reaching healthcare workers in far‐flung areas. These e‐learning platforms offer modules on climate health, enabling asynchronous learning and empowering nurses to stay updated with evolving climate‐related health risks (Hassanain, 2017).

When it comes to patient and community education, nurses transition from caregivers to educators. Recognizing that knowledge is the first line of defence, they engage in community outreach programs, disseminating information on climate‐related health risks, preventive measures and emergency preparedness. This includes everything from teaching communities about the health risks of heatwaves to guiding them on mosquito control measures during rainy seasons to curb vector‐borne diseases. Tailored communication strategies, which respect local cultures, languages and practices, ensure that these educational messages are both accessible and actionable (Alshuwaikhat & Mohammed, 2017).

In line with community education, many nurses have also initiated community‐based projects, promoting environmental sustainability. Through endeavours such as community gardens or recycling initiatives, they emphasize the interconnectedness of health, environment and climate, driving home the message that every individual has a role to play in mitigating climate change and its health impacts (Alomair, 2015).

Advocacy, a more proactive arm of the nursing profession, sees nurses amplifying their voices in favour of robust health policies and strategies. They engage with policymakers, lobby for increased funding in climate‐health research and influence healthcare strategies to be more resilient in the face of changing climatic conditions. By representing the healthcare community in global climate summits and local policy dialogues, nurses ensure that health remains at the forefront of climate discussions. Their testimonies, rooted in firsthand experiences and patient stories, add a human touch to the otherwise data‐heavy climate debates, compelling decision‐makers to act (Alshahrani, 2020).

Furthermore, many nurses join or form coalitions with environmental activists and organizations. These partnerships accentuate the health‐centric viewpoint in larger environmental campaigns, underlining the direct health repercussions of climate change. Such collaborations also provide a platform for nurses to share their field experiences, further adding authenticity and gravitas to the broader climate change dialogue. These firsthand accounts, detailing the tangible effects of climate change on patient health, resonate strongly with the public, making the abstract concept of global warming more immediate and personal. Through these alliances, nurses not only amplify their advocacy efforts but also foster interdisciplinary collaborations, creating a more cohesive and holistic response to the multifaceted challenges posed by climate change (Sheikh et al., 2019).

In the digital age, many nurses are integrating technology into their advocacy and education endeavours. By utilizing platforms such as telehealth, webinars and mobile health applications, they can reach wider audiences, offering climate health‐related advice and resources. These platforms also serve as interactive forums where communities can share their experiences, seek guidance and collaborate on grassroots initiatives, further reinforcing the preventative measures against climate‐induced health risks (El Mallakh, 2015).

Nurses, given their close ties to the communities they serve, often possess deep cultural insights. They understand the nuances and intricacies of local beliefs, customs and traditions. This cultural sensitivity translates into more effective advocacy, as nurses can frame climate change narratives in ways that resonate with local sentiments and values. By intertwining climate change concerns with cultural narratives, they foster a deeper, more personal connection between communities and the larger global issue, encouraging more proactive local engagement (Almaghaslah & Alsayari, 2020).

The triad of capacity building, education and advocacy underscores the multifaceted role nurses play in addressing climate change and its health impacts. By donning multiple hats—as trainers, educators and advocates—they champion a holistic approach, ensuring that the healthcare community is both reactive and proactive in its response to the evolving climate challenge (El Mallakh, 2015).

### 3.5 Disaster preparedness and emergency response

The intensifying effects of climate change have amplified the frequency and severity of natural disasters, placing an unprecedented strain on healthcare infrastructures worldwide. Amidst this landscape, nurses' roles have evolved, necessitating them to not only provide care but also be adept at disaster management, ensuring that communities can swiftly and effectively respond to these challenges.

Disaster preparedness encompasses a suite of skills that enables nurses to anticipate, prepare for and respond to the health impacts of climate‐induced emergencies. From understanding the nuanced health implications of various disasters to orchestrating mass casualty management, nurses play a pivotal role in mitigating the impacts of these emergencies. They participate in routine disaster drills, honing their skills in triage, mass casualty management and psychological first aid. These simulations ensure that, when real disasters strike, nurses are not only technically proficient but can also manage the psychological and emotional toll that such events often entail (El Mallakh, 2015).

The rapid advancement in technology has ushered in novel ways for nurses to prepare for and manage disasters. The integration of Geographic Information Systems (GIS) in healthcare allows nurses to anticipate vulnerable areas in the face of impending natural disasters, facilitating pre‐emptive evacuations and resource allocations. Mobile health applications, which provide real‐time health advisories during disasters, have become instrumental tools in the hands of nurses. They enable them to offer remote consultations, track disease outbreaks and monitor resource levels in healthcare centres, ensuring that aid is directed where it's most needed (Al Garni & Awasthi, 2017).

In the event of actual disasters, nurses are frequently part of frontline response teams. Their ability to function efficiently under pressure becomes paramount, as they provide immediate medical care, manage emergency medical supplies and ensure the continuity of essential health services. Their role often extends beyond immediate clinical care. They act as liaisons between affected communities and broader healthcare systems, facilitating effective communication and ensuring that resource allocation aligns with on‐ground needs (Alrehaili, 2021).

It's imperative to recognize that disaster management is a collective endeavour. Nurses often collaborate with other professionals such as paramedics, emergency medical technicians and public health experts to create a unified front in disaster response. Through interdisciplinary teamwork, these professionals devise comprehensive strategies that leverage each profession's unique strengths, ensuring a holistic approach to disaster management. Regular interprofessional training sessions and workshops reinforce this collaborative spirit, enhancing coordination and improving overall response efficiency during actual emergencies (Alyami et al., 2020).

Moreover, the intricate understanding nurses possess regarding their local communities aids in providing culturally competent care, ensuring that interventions are not just medically appropriate but also culturally sensitive. This is particularly crucial in regions with diverse populations, where traditional beliefs and practices can influence health‐seeking behaviours during disasters (AlQahtany & Abubakar, 2020).

While the immediate physical health implications of disasters are evident, the long‐term mental health consequences cannot be overlooked. Nurses, with their close patient interactions, are often the first to identify signs of post‐traumatic stress disorder, anxiety or depression following disasters. They play a critical role in facilitating psychological interventions, offering initial counselling and referring affected individuals to specialized mental health professionals. Ensuring mental well‐being is paramount for community recovery and resilience, and nurses are at the forefront of this endeavour, bridging the gap between disaster aftermath and psychological healing (Bajow et al., 2015).

As climate change continues to reshape our global landscape, nurses stand as pillars of resilience in the face of these adversities. Their expertise in disaster preparedness and emergency response, combined with their unwavering commitment to patient care, ensures that communities are not only equipped to withstand climate‐induced emergencies but can also bounce back, embodying the true spirit of resilience (Alharbi et al., 2020).

### 3.6 Research, adaptation and evidence‐based practice

In an age where climate change continually poses new and dynamic health challenges, the importance of research and evidence‐based practice in nursing cannot be understated. The ability to collate data, interpret it and implement effective interventions is pivotal in ensuring that healthcare systems remain resilient and responsive.

One primary aspect is the cultivation of research competencies among nurses. Nurses are uniquely positioned to observe firsthand the direct and indirect health impacts of climate change on their patients. This grassroots insight, combined with a structured approach to data collection, allows for the generation of research that is not only relevant but also actionable. This involves identifying prevalent health issues within their communities, conducting surveys and even participating in larger epidemiological studies (Bashiri et al., 2021).

In the digital era, the utilization of digital health tools has become increasingly prevalent in the nursing profession. From electronic health records to wearable health monitors, the abundance of data generated can be instrumental in climate change‐related health research. Nurses are often at the forefront of leveraging this data, analysing patterns and drawing correlations between environmental factors and health outcomes. By integrating digital insights with traditional research methodologies, nurses can paint a more comprehensive picture of the climate‐health nexus, driving evidence‐based interventions with a blend of technology and human touch (Amer et al., 2019).

Adapting to the ever‐evolving challenges of climate change requires a fluid approach to healthcare. Evidence‐based practice ensures that nursing interventions are not based on intuition alone but are grounded in rigorous scientific research. The integration of research findings into daily nursing practice ensures that patient care is both current and effective. Nurses engage in continuous learning, participating in workshops and training programs that equip them with the latest in healthcare research and best practices (Hasani et al., 2020).

The multifaceted challenges of climate change necessitate a multidisciplinary approach to research and adaptation. Nurses frequently collaborate with environmental scientists, public health experts and social scientists to conduct comprehensive studies. Such interdisciplinary collaborations bring varied perspectives, enriching the depth and breadth of research. By combining nursing insights with expertise from other fields, more holistic solutions to climate‐induced health challenges can be formulated, ensuring that interventions are both scientifically sound and practically feasible (Alqahtani et al., 2018).

Furthermore, in the context of Saudi Arabia, where rapid urbanization, cultural nuances and unique climatic conditions converge, tailored healthcare strategies are imperative. Evidence‐based practice becomes especially crucial. Nurses, through their deep‐rooted community ties and understanding of local health dynamics, can refine and adapt global best practices to fit the specific needs of the Saudi populace. This involves not just clinical care adjustments but also community outreach, patient education and advocacy to ensure holistic health outcomes (Alshehri et al., 2019).

In regions like Saudi Arabia, where ancient traditions and modern science often converge, the value of traditional knowledge in healthcare adaptation cannot be overlooked. While evidence‐based practice remains paramount, nurses often find value in integrating traditional healing methods and practices into their care regimens. By combining time‐tested traditional practices with modern research, nurses can offer care solutions that resonate with the local populace's beliefs and values, enhancing patient trust and ensuring better health outcomes (Babiker Mohamed et al., 2022).

The nexus between research, adaptation and evidence‐based practice in nursing stands as a testament to the profession's commitment to excellence. As nurses navigate the challenges posed by climate change, their reliance on scientific research, coupled with their adaptability, ensures that healthcare remains a beacon of hope, especially in regions like Saudi Arabia that face unique challenges (Alduraywish et al., 2021).

### 3.7 Cultural, ethical and regional considerations

Nursing, at its core, is not merely a profession that deals with the physiological aspects of patient care; it encompasses the holistic understanding of patients, including their cultural, ethical and regional backgrounds. Especially in the context of climate change, where reactions and perceptions can be deeply intertwined with these aspects, nurses play a pivotal role in bridging healthcare and cultural understanding.

Saudi Arabia, with its rich tapestry of traditions, presents unique challenges and opportunities for healthcare professionals. Historically, the Kingdom has always placed immense value on traditions and customs, which significantly influence health behaviours and perceptions (Hamoudah et al., 2021). Nurses, therefore, must possess a deep understanding of these cultural nuances to ensure that their interventions resonate with the local populace. For instance, approaching health education campaigns about climate change impacts might need to be curated differently, keeping in mind local traditions and beliefs (Baig et al., 2019).

In Saudi Arabia, where spirituality and religion are interwoven with daily life, nurses must recognize and respect the spiritual beliefs of their patients. Understanding the significant role of faith in the Saudi society can aid nurses in providing compassionate care, especially in the face of climate‐related anxieties and health challenges. Engaging in conversations about spirituality, offering spaces for prayer and incorporating religious rituals in patient care can provide solace and enhance the overall wellbeing of patients, making them more resilient to climate‐induced stresses (Allbed et al., 2017).

Beyond cultural considerations, ethical aspects in nursing care are paramount. Climate change can exacerbate health disparities, with the most vulnerable often being the hardest hit. Ensuring equitable care, without any form of discrimination, becomes an ethical imperative for nurses. They must champion the cause of marginalized groups, ensuring that they receive the requisite care and support in the face of climate‐induced health challenges (Cruz et al., 2018).

As climate change impacts intensify, the potential for resource shortages becomes a grim reality. Nurses might be confronted with ethical dilemmas related to resource allocation, especially in emergency situations exacerbated by climate events. Training in ethical decision‐making and establishing clear guidelines can equip nurses to navigate such situations, ensuring that care is dispensed judiciously and with utmost integrity (Alotaibi et al., 2018).

Regional specificities of Saudi Arabia, such as its desert climate, urbanization patterns and socio‐economic dynamics, further complicate the health impacts of climate change. For instance, water scarcity, exacerbated by climate change, can lead to unique health challenges in the region, requiring tailored nursing interventions. Additionally, rapid urbanization might lead to lifestyle‐related diseases, which, combined with climate change impacts, require a dual strategy for effective nursing care (Koehrsen, 2021).

Saudi Arabia is home to nomadic Bedouin communities, who have a unique lifestyle deeply rooted in the desert environment. The impacts of climate change, such as shifting sand dunes and altered migration patterns, can pose distinct health challenges to these communities. Nurses working in or for these communities need to be aware of their unique lifestyle, health beliefs and challenges to provide tailored care that addresses both their immediate and long‐term health needs.

A successful nursing strategy in Saudi Arabia, especially in the context of climate change, cannot be a one‐size‐fits‐all approach. It needs to be deeply rooted in understanding the cultural, ethical and regional dynamics of the Kingdom. Such an approach not only ensures effective patient care but also strengthens the bond of trust between nurses and the communities they serve (Howarth et al., 2020).

## 4 DISCUSSION

The profound impacts of climate change on global health have prompted a surge in studies aiming to understand its various facets. The nexus of climate change and nursing in Saudi Arabia, as discussed in this review, presents a captivating paradigm, illustrating the intricate interplay of environmental shifts, healthcare dynamics and socio‐cultural aspects. The focus on Saudi Arabia offers a unique perspective, given the country's specific geographical, cultural and developmental considerations.

Central to our findings is the understanding of climate change and its ramifications on health, a notion highlighted in previous global studies (Wu et al., 2016). This foundational knowledge acts as the linchpin for all subsequent nursing actions. From heatwaves to alterations in air and water quality, nurses in Saudi Arabia are at the forefront of addressing the immediate health impacts of these climatic challenges. A study conducted in Africa showcased similar challenges, wherein the role of nurses in educating communities about heat‐related illnesses became paramount, reiterating our results (Kurth, 2017).

Addressing both direct and indirect health implications requires multifaceted nursing interventions. This isn't just a phenomenon restricted to Saudi Arabia. For example, research in Bangladesh highlighted nurses' roles in not only managing heatstroke patients but also in advocating for infrastructure improvements to combat urban heat islands (Abrar et al., 2022). Likewise, our review underscores the importance of such interventions tailored to the Saudi context, emphasizing infrastructure and health system adjustments to handle climate change's cascading effects.

Furthermore, vulnerable populations, often bearing the brunt of climate adversities, necessitate specialized care. Our identification of such groups aligns with global sentiments, where similar population subsets, from pregnant women in flood‐prone Bangladesh to the elderly during European heatwaves, needed tailored healthcare solutions (Chisty et al., 2021). Saudi Arabia's remote communities, and its unique societal dynamics, bring about distinct challenges that necessitate both cultural sensitivity and clinical expertise.

The efforts made to overcome these challenges include training programs for nurses focused on climate‐related health issues, community outreach initiatives and the development of emergency preparedness plans tailored to the unique needs of Saudi Arabia's diverse population. Our research offers novel insights by proposing a specific framework for enhancing nurse competencies in Saudi Arabia, which can be adapted to similar contexts globally.

One area that demands more attention is the psychosocial impact of climate change on populations. Our discussions centred around Saudi Arabia hint at the potential mental health challenges that might arise due to extreme weather events or displacement. This observation finds resonance in global studies as well. For instance, research from Scandinavian countries reveals an increase in anxiety and mood disorders linked to climate‐related stresses (Sangervo et al., 2022). Nurses, especially psychiatric nurses, thus have a significant role in offering counselling, facilitating support groups and ensuring mental well‐being in the backdrop of climate adversities.

In the realm of capacity building and advocacy, our findings resonate with global healthcare initiatives. The World Health Organization, for instance, underscores the significance of training healthcare professionals about climate change, a tenet echoed in our results pertaining to Saudi Arabia (Jobran, n.d.). Advocacy, particularly in policy formulation, is another domain where nurses can leave an indelible mark, as seen from proactive nursing communities in countries like Australia championing climate‐smart healthcare policies (National Council of State Boards of Nursing, 2021).

The importance of integrating climate change topics within nursing education cannot be overemphasized. Our findings pertaining to Saudi Arabia, when juxtaposed with global trends, showcase a definitive shift in nursing curricula. For instance, institutions in Europe have begun infusing climate change and health modules within their nursing programs, emphasizing the importance of being climate‐literate in today's world (Skela‐Savič et al., 2020). This not only equips nurses with the requisite knowledge to tackle climate‐induced health challenges but also instils a sense of environmental stewardship, propelling them to advocate for sustainable practices in healthcare.

Our spotlight on disaster preparedness and emergency response aligns with global emergency nursing frameworks, especially from regions prone to climate disasters like the Pacific Islands. Here, nurses undergo specialized training to handle cyclones and flooding, mirroring the preparedness aspects discussed in our review for Saudi Arabia (Rameshshanker et al., 2021).

Research and evidence‐based practice in nursing, as emphasized in our findings, remain universally recognized tools to combat climate change health impacts. Parallel findings from Asian nations indicate that nurses are increasingly being roped into research activities, collecting valuable data and tailoring interventions in light of such evidence (Pun et al., 2018). The specificity of the Saudi context amplifies the need for region‐centric research, offering solutions that resonate with the nation's unique landscape and challenges.

The rapid advancements in technology, especially in the healthcare domain, present promising avenues to address climate change's health ramifications. Our review hints at the importance of digital health platforms in managing climate‐related health crises in Saudi Arabia. This is a sentiment shared globally, with telemedicine platforms in regions like South America providing remote consultations during flood events or tech‐enabled early warning systems in Southeast Asia alerting communities about impending heatwaves (Reguero et al., 2015). These technological interventions, when tailored to specific regional needs, can play a pivotal role in mitigating climate change's health impacts.

A salient feature of our study was the emphasis on cultural, ethical and regional considerations in nursing care related to climate change. Culturally tailored care, with an understanding of ethical obligations, stands as a beacon in holistic healthcare delivery. A comparative reflection can be found in studies from Canada, where indigenous communities are prioritized, and interventions are tailor‐made to resonate with their cultural and traditional nuances, emphasizing the universality of this principle (Généreux et al., 2019).

The multifaceted challenges of climate change necessitate a multidisciplinary approach to healthcare. Our review showcases the pivotal role of nurses in Saudi Arabia in this regard. However, collaborating with other healthcare professionals, urban planners, environmentalists and policymakers further strengthens intervention strategies. A study from Japan elucidates this collaborative model, where nurses work alongside city officials and architects to design climate‐resilient healthcare infrastructures (Yang et al., 2023). Such synergistic efforts amplify the efficacy of interventions and create a holistic response to climate change.

As climate change continues its relentless march, its health implications magnify in tandem. Our review, focusing on the Saudi Arabian context, serves as a testament to the monumental role nurses play in this evolving narrative. Their responsibilities, ranging from immediate healthcare delivery to long‐term policy advocacy, set the tone for proactive healthcare in the face of environmental adversities.

A noteworthy aspect emerging from our discussion is the role of nursing leaders in driving climate action. The nursing profession, by virtue of its widespread reach and trust, can galvanize communities and policymakers alike. This is not unique to Saudi Arabia. For instance, nursing associations in New Zealand have been at the vanguard of climate protests, advocating for robust policies and sustainable practices (Kotcher et al., 2021). Such leadership endeavours not only bring about tangible policy changes but also inspire younger nursing cohorts to take up the mantle of climate advocacy.

In parallel with direct care, an important consideration is the environmental footprint of healthcare establishments. Our focus on Saudi Arabia brought forth the idea of resource conservation within healthcare settings. Globally, there's a burgeoning movement advocating for ‘green healthcare’ (Khan et al., 2022). Hospitals in Germany, for instance, are transitioning to renewable energy sources and implementing waste reduction programs, with nurses often being the champions of these sustainable initiatives (Godbole & Lamb, 2018). Such endeavours not only mitigate the environmental impact of healthcare but also serve as exemplars for the community at large (Barnes, 2020; Nicholas & Breakey, 2017; Setoguchi et al., 2022).

Drawing parallels with global scenarios reinforces the universal nature of these challenges and the consequent solutions (Goniewicz, Khorram‐Manesh, & Burkle, 2023; Hathaway & Maibach, 2018; Maxwell & Blashki, 2016; McDermott‐Levy et al., 2019; McNamara & Des Combes, 2015; Perumal, 2018; Rocque et al., 2021; Trombley et al., 2017). While each region has its idiosyncrasies, the core principles of nursing care remain steadfast across borders. It's heartening to note that, despite the vast cultural and geographic differences, the global nursing community shares a collective goal and vision in tackling climate change's health implications.

The research highlights the critical need for tailored nursing competencies to address climate change impacts in Saudi Arabia. The proposed framework emphasizes the importance of continuous education, interdisciplinary collaboration and cultural sensitivity. These findings not only provide a roadmap for enhancing nurse preparedness in Saudi Arabia but also offer valuable insights for global health strategies.

This discussion sheds light on the multifaceted dimensions of nursing in the context of climate change, emphasizing both the unique aspects of the Saudi Arabian context and the universal principles guiding nursing care globally. Future research endeavours should delve deeper, widening the scope and collaborating internationally, to chart out holistic strategies that encompass the vast spectrum of challenges and solutions in this domain.

## 5 LIMITATIONS

This rapid review provides vital insights into the nexus between climate change and nursing within the Saudi Arabian context, but several limitations should be acknowledged. Due to the time‐sensitive nature of rapid reviews, our methodology prioritized speed, which may have resulted in overlooking some pivotal studies. This inherent trade‐off between timeliness and comprehensiveness should temper interpretations of our findings.

Our choice of databases and search criteria might introduce selection bias. While our intent was thoroughness, the potential omission of key research articles remains. The methodology's swiftness may not permit an exhaustive exploration of the patient's nuanced facets, a depth more achievable with systematic reviews.

There might also be variability in the selection and interpretation of studies if multiple reviewers were engaged. The quality assessment of included studies might not have been as exhaustive as required, presenting potential biases. The omission of grey literature—such as reports, conference abstracts and theses—could also limit the review's scope.

Focusing primarily on Saudi Arabia restricts the generalizability of our findings to regions with disparate socio‐cultural and geographical contexts. Moreover, while we endeavoured to encompass a wide array of sources, linguistic constraints might have curtailed our exploration of significant regional studies published in non‐English mediums.

## 6 CONCLUSIONS

Climate change, with its formidable challenges, beckons healthcare systems worldwide to respond proactively. At the heart of this global health challenge is the nursing profession. Our review elucidates the expansive and multifaceted roles nurses undertake in mitigating and managing the health implications of these environmental shifts in Saudi Arabia.

The research findings indicate that effective nursing strategies lie in a profound understanding of climate change and its myriad health ramifications. Nurses in Saudi Arabia must address both direct impacts, such as heatwaves, and indirect effects on air and water quality.

The nuanced needs of vulnerable populations, such as pregnant women, the elderly and socio‐economically marginalized groups, demand targeted nursing interventions. These strategies must be tailored to Saudi Arabia's unique cultural, ethical and geographical facets.

Interdisciplinary collaborations are crucial for success in addressing climate‐induced health challenges. Nurses, in tandem with other healthcare professionals, policymakers, environmentalists and community leaders, can forge a united front against these adversities.

Nursing education must integrate climate change topics to equip nurses with the necessary knowledge and skills. Continuous learning and the use of digital health tools will enhance preparedness and response to climate‐related health challenges.

The leadership role of nurses in driving climate action is essential. By advocating for robust policies and sustainable practices, nurses can significantly influence community and policy decisions.

As the globe confronts unprecedented health challenges catalysed by climate change, nurses stand as pillars of resilience and innovation. Their dexterity, spanning clinical care to advocacy, renders them indispensable in this global endeavour. This review aims to galvanize further research, drive policy innovations and catalyse comprehensive interventions, forging a path towards a healthier, more resilient future in the face of climate uncertainties.

## AUTHOR CONTRIBUTIONS

Z.A.M. provided the main framework, identified and organized primary materials, and collaborated in writing the manuscript. K.N. identified the appropriate references and collaborated in writing and reviewing the paper. K.G. identified appropriate references and collaborated on the writing and editing of the manuscript. All authors have read and agreed to the published version of the manuscript.

## FUNDING INFORMATION

This research received no external funding.

## CONFLICT OF INTEREST STATEMENT

The authors declare no conflicts of interest.

## Data Availability

Data available on request from the authors.
